# The Distribution and Genetic Variability of Potato Viruses in Russian Regions

**DOI:** 10.32607/actanaturae.27830

**Published:** 2026

**Authors:** V. O. Samarskaya, F. A. Butyrin, T. P. Suprunova, N. A. Spechenkova, M. E. Taliansky, N. O. Kalinina

**Affiliations:** Shemyakin–Ovchinnikov Institute of Bioorganic Chemistry, Russian Academy of Sciences, Moscow, 117997 Russia; Doka-Gene Technologies Ltd., Rogachevo, Dmitrovsky District, Moscow Region, 141880 Russia; Belozersky Institute of Physico-Chemical Biology, Lomonosov Moscow State University, Moscow, 119234 Russia

**Keywords:** potato, potato viruses, RNA sequencing, viral genome, de novo assembly, phylogeny, genetic diversity, regions of the Russian Federation

## Abstract

Viral diseases represent an increasingly serious threat for potato production all around the world, including in the Russian Federation, which leads to a significant decrease in potato crop yield, quality, and shelf life. In this study, we carried out screening of potato leaf and tuber samples collected from commercial potato fields to determine the spread of potato viruses in 16 regions of the European part, and two regions in the Ural Federal District, of the Russian Federation. The samples were sequenced, and full-length viral genomes were subsequently assembled *de novo*. A phylogenetic analysis of the identified virus variants was performed to assess their genetic diversity and possible origin. It has been shown that the most dangerous and economically important potato virus Y (PVY) is widespread and is represented by recombinant variants, NTNa, NTNb, and N-Wi being the most common ones. The second most common virus was potato virus M (PVM), which was frequently encountered in conjunction with potato virus S (PVS). The presence of potato leafroll virus (PLRV), which is recognized as an economically detrimental potato pathogen, along with PVY, has been found in two Russian regions. Mixed infections were detected in at least half of the studied samples, many containing both PVY and PVM (about one-third of all the samples). The data on the evolutionary variability of virus populations lay the groundwork for developing innovative strategies meant to contain a broad range of viruses and their strains using specifically designed double-stranded RNA (dsRNA).

## INTRODUCTION

Potato (*Solanum tuberosum *ssp. *tuberosum *L.) is the fourth most important food crop in the world (after rice, wheat, and maize) and the most important non-cereal crop. Annual global potato production stands at over 390 million tons (FAOSTAT Database). In the Russian Federation, potato has traditionally been one of the main agricultural crops (the so-called “second bread”), its annual production being ~ 30 million tons. Viral diseases affecting potato pose a serious threat to global agriculture, reducing the crop yield, quality, and shelf life of the product. During viral epidemics, crop yield loss can rise as high as 50% or even more [1, 2, 3], negatively impacting food security and economic stability. More than 40 viruses that naturally attack potato have been reported; however, only nine of those are of significant economic relevance to global potato production. These are Potato leafroll virus (PLRV); the Potato viruses A, M, S, V, X, and Y (PVA, PVM, PVS, PVV, PVX, and PVY, respectively); Potato mop-top virus (PMTV); and the Tobacco rattle virus (TRV). All these viruses share a single-stranded RNA (ssRNA) genome. The viruses are transmitted via insect vectors (PVY, PLRV, PVM, and PVS), through tubers, and by mechanical damage. PVY, PVS, PVM, PLRV, and PVX are the main pathogens responsible for crop yield loss and deteriorating potato quality [2, 4, 5]. Quarantine agents that are present in elite and high-reproduction seed potatoes in the Russian Federation include eight potato viruses: PLRV, PVA, PVM, PVS, PVX, PVY, PMTV, and TRV. The economic damage from the infection of potato plants by the aforementioned viruses varies depending on the virus strain, the presence of mixed infections, vector activity, region (geographical area), climatic conditions, and the overall level of agricultural technological development [2, 4, 5, 6].

Monitoring the spread of plant viruses and their novel variants resulting from genetic evolution, transmission from natural reservoir plants, mixed infections, changes in agricultural practices, and global warming is required in order to combat viral epidemics and develop modern methods to protect agricultural plants against viral infections [3, 5]. Certification schemes for seed material have been developed to address this problem, and they are implemented by specialized laboratories that perform diagnostics and sanitation of potato cultivars. Potato is also bred for resistance to viral infections. Pesticides are used to control insect vectors of viral infections. Generating potato lineages/cultivars resistant to viral infections using bioengineering technologies, the main one being CRISPR-Cas genome editing, has now become a consequential undertaking in the development of highly efficient potato production. An alternative method for protecting plants against viruses is an intensely developing innovative strategy based on the RNA interference mechanism. It utilizes exogenous dsRNAs specific to the nucleotide sequence of the viral genome [7].

Only a few studies describing strains/isolates of potato viruses, as well as their distribution across the Russian Federation, have been published so far [8, 9, 10, 11]. Thus, a high incidence of potato infection with PVM, PVS, and PVY has been detected in the European part of the Russian Federation and the Irkutsk region. The potato viruses PVM, PVS, PVY, PVX, and PLRV have been detected in the leaves and tubers of potato samples collected in the Northwestern, Volga, and Far Eastern Federal Districts of the Russian Federation [11]. PVY, PVM, and PVS were found to be the viruses most frequently infecting potato plants in the Novosibirsk region as well [12]. Molecular characterization of the Potato virus P detected in Primorsky Krai revealed that it is a distinct Russian isolate [10, 11].

ELISA techniques using virus-specific antibodies and reverse transcription polymerase chain reaction (RT-PCR) are the key techniques used for analyzing potato viral infections and characterizing virus strains and isolates. Therefore, phylogenetic analysis of virus strains is generally based on nucleotide sequences encoding individual virus-specific proteins, primarily coat proteins. Next-generation sequencing has recently appeared as a tool in the diagnosis and investigation of the composition of the virus population [13, 14, 15]. In the present study, we employed this method to perform RNA sequencing from potato leaves and tubers (RNA-Seq), followed by *de novo *assembly of full-length viral genomes, in order to identify the major economically important potato viruses in 18 regions of the Russian Federation different in terms of climatic conditions, reservoir plants, and vector species. The results of the analysis of the data obtained for the material collected in 2021–2024 are reported. This study aimed to acquire both data on the distribution of potato viruses in individual regions of the Russian Federation and the whole-genome sequencing data for these RNA viruses, which are characterized by a high level of recombination and point mutations. We analyzed RNA from randomly collected leaf samples of first-generation plants and high-reproduction seed potatoes (super-super-elite and super- elite) grown on commercial fields, as well as from the leaves of tuber seedlings of these plants. PVY, PVM, and PVS were shown to be the main viruses infecting potato plants during the field season. The PVM and PVS were encountered only in some regions, whereas various recombinant variants of PVY circulate ubiquitously, including the so-called rare and novel recombinants, including the ones described previously by us [16]. PLRV was also detected in certain regions. Importantly, the most prominent and economically fraught viruses, such as PVY and PLRV, were detected in potato tubers. Not only are our findings important for elaborating measures to contain the spread of potato viruses based on monitoring of their geographic distribution, but they are also used to develop methods based on dsRNA molecules specific to viral genome sequences to ensure bioprotection of potato against viruses by triggering the RNA interference mechanism in plants, which eliminates viral RNA.

## EXPERIMENTAL

**Plant sample selection**

Potato (*Solanum tuberosum *L.) plants were used as the study objects. Leaf samples and tubers were collected on commercial agricultural fields in 2021–2024 in different regions of the Russian Federation. Samples of 14 potato cultivars different in their susceptibility to viral infections were collected in 18 Russian regions: the Northwestern Federal District – Pskov and Novgorod regions; Central Federal District – Moscow, Bryansk, Oryol, Tver, Kostroma, Yaroslavl, Vladimir, and Tambov regions; Volga Federal District – Nizhny Novgorod, Penza regions and the Republic of Tatarstan; North Caucasian Federal District – Stavropol Krai; Southern Federal District – Astrakhan region and Krasnodar Krai; and Ural Federal District – Sverdlovsk and Tyumen regions.

Leaves were sampled 2 or 3 months after planting tubers in the field. Samples were collected randomly, evenly across the planting area, without any bias based on external plant characteristics. Material (samples) was collected from an average of 25–60 plants for each cultivar/genotype. In order to preserve plant tissue, leaf punches were collected into tubes containing RNA fixative (Eurogen, Russia). Tubers were harvested at the end of the growing season and stored for 3–5 months at +4°C (30–60 tubers per cultivar). After the dormancy period, an explant containing the apical meristem (the terminal bud) was excised from each tuber. The scheme “one tuber – one explant – one seedling” was employed. The explants were airdried for 24 h and then planted in boxes filled with peat. Seedlings were grown under controlled greenhouse conditions (protected from insect access; temperature, 21–22°C) for 3–4 weeks until the 5–8 leaf stage. The sample for analysis contained leaf punches from 30–60 seedlings.

**RNA isolation and high-throughput sequencing**

Plant tissue samples (leaf punches from field-grown plants and the leaves of tuber seedlings) were frozen in liquid nitrogen and homogenized in a mortar with a pestle to a powder consistency. Total RNA was extracted using the TRIzol reagent (InvitrogenTM TRIzolTM Reagent, ThermoFisher ScientificTM, USA) according to the manufacturer’s protocol. The resulting RNA samples were dissolved in nuclease-free water (NFW, Thermo Fisher ScientificTM, USA) and treated with DNase I (RNase-free DNase I, Thermo #EN0523, USA). Next, RNA was re-precipitated using TRIzol-chloroform. The quantity and quality of the extracted RNA were assessed using a NanoDrop ND-1000 spectrophotometer (Nanodrop Technologies, USA) and non-denaturing agarose gel (1.5%) electrophoresis.

The quality assessment of the RNA samples (determining the RNA integrity number (RIN) for each sample), library preparation for sequencing, and sequencing were performed at the research facilities of CeGaT GmbH (Tübingen, Germany). RNA-seq libraries, including rRNA depletion, were prepared using TruSeq Stranded Total RNA in combination with the Ribo-Zero kit (Illumina, USA). The prepared libraries were sequenced using an Illumina NovaSeq 6000 system with PE100 parameters (2 × 100 bp paired-end reads). A total of 113 samples (49 leaf and 64 tuber samples) were analyzed.

The resulting 113 RNA-seq libraries contained from 2,701,505,437 to 12,600,931,349 paired-end reads (2 × 100 bp) at Q30 values ranging from 84.43% to 95.88%. Demultiplexing was performed using bcl- 2fastq v2.20 (Illumina); adapter trimming was carried out with Skewer v0.2.2; and read quality control was performed using the FastQC v0.11.9 and SeqKit v2.3.0 tools.

**Processing and analysis of the RNA sequencing data**

*De novo *assembly of the virus genomes was performed using the Trinity v2.15.2 [17] and rnaviral- SPAdes v3.15.4 [18] algorithms. For reference-guided assembly, the samples were analyzed for the potato viruses PVY, PVX, PVS, PVM, PLRV, PVA, PVP, TMV, PVT, PVV, TNV, PRDV, PAMV, AMV, TRV, and PMTV using the HISAT2 v2.1.0 [19], samtools, and bcftools software. We identified contigs containing full-length genomic RNA sequences: PVY – 158 contigs; PVS – 27 contigs; PVM – 127 contigs; PLRV – 6 contigs; and one contig with the full-length PVP genome sequence. These contigs were selected for further analysis and separated from the remaining contigs in the assemblies. Translation of the assembled nucleotide sequences was performed using the NCBI ORFfinder v0.4.3 tool [20]. All the full-length contigs contained open reading frames characteristic of these viruses. Taxonomic classification of nucleotide and translated amino acid (protein) sequences was performed using the BLAST algorithm (BLASTn v2.14.0+) and the NCBI database [21]. For assessing the assembly quality and correcting potential errors, raw reads were mapped to the resulting assembly. Mapping was performed using HISAT2 v2.1.0 [19]. The assemblies were checked for single-nucleotide errors, short insertions, deletions, and gaps using Tablet v1.21.02.08 [22].

Multiple nucleotide sequence alignments were performed using the MAFFT software (v7.453) set on default parameters. Poorly aligned and hyperdivergent regions were eliminated using the Gblocks software (v0.91b) set on default settings, followed by concatenation of the resulting fragments [23]. The 5′ and 3′ terminal regions of the sequences were trimmed to generate alignment of full-length coding sequences. A maximum-likelihood phylogenetic analysis was conducted using the IQ-TREE software (v1.6.12) employing 1,000 bootstrap replicates to assess statistical significance and automatic model selection. Most clusters demonstrate high support levels (> 87.5), confirming the reliability of the topology obtained. Tree visualization was performed using the iTOL v7.2.1 tool [24].

The sequences of the selected contigs and the libraries have been deposited in the NCBI database under access number PRJNA13227266. *Supplementary Table 1 *summarizes the data on the samples and RNA-seq libraries.

**Statistical analysis**

Statistical analysis was conducted using Pearson’s χ² test to probe for differences in the mixed infection rate depending on a certain year and region. At expected rates < 5 cases, the categories were merged and the statistical significance level was set at *p* < 0.05.

## RESULTS AND DISCUSSION

**The key potato viruses detected in the Russian regions**

**Fig. 1:**
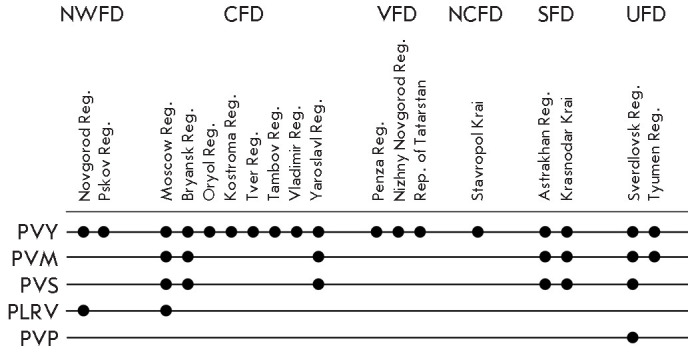
The geographic distribution of potato viruses in the Russian regions. The Russian regions are grouped by Federal Districts: NWFD – Northwestern, CFD – Central, VFD – Volga, NCFD – North Caucasian, SFD – Southern, and UFD – Ural Federal Districts. Colored lines indicate the viruses: PVY (Potato virus Y), PVM (Potato virus M), PVS (Potato virus S), PLRV (Potato leafroll virus), and PVP (Potato virus P). Dots represent the regions where the corresponding viruses were detected

Monitoring of viral diseases affecting potato conducted in several regions of the Russian Federation revealed significant differences in the incidence rates of various viruses. We identified a total of five potato viruses: PVY, PVM, PVS, PLRV, and PVP (*Fig. 1*). Potato virus Y (PVY) was found to be the most ubiquitous as it was present in all the studied regions, which is confirmation of its high epidemiological significance. Potato virus M (PVM) was the second most frequently detected virus, found in the Central Federal District (Moscow, Yaroslavl, and Bryansk regions), the Southern Federal District (Astrakhan region and Krasnodar Krai), and the Ural Federal District (Sverdlovsk and Tyumen regions). Potato virus S (PVS) was detected in the same regions as PVM, except for the Tyumen region. Potato leafroll virus (PLRV) was revealed only in the Moscow region (Central Federal District) and the Novgorod region (Northwestern Federal District), attesting to the limited distribution of this virus. Potato virus P (PVP) was detected only in the Sverdlovsk region (Ural Federal District). Neither Potato virus X nor Potato spindle tuber viroid was detected in any of the analyzed samples. Hence, our findings on the highest incidence rates of PVY, PVM, and PVS are consistent with the data obtained previously in the studied Russian regions [8, 9, 10, 11, 12] and are indicative of the broad geographic variability in the distribution of different potato viruses; PVY exhibited the best adaptability and resistance to climatic and geographical conditions.

Mixed infections (more than one virus per sample) were detected in 50% of the samples (56 samples out of 113). Individual viruses present within mixed infections were distributed as follows: PVM, in 51 out of 56 samples (91%); PVY, in 47 out of 56 samples (84%); PVS, in 15 out of 56 samples (27%); PLRV, in 5 out of 56 samples (9%); and PVP, in one out of 56 samples (2%). The following virus combinations were identified in mixed infections: PVM + PVY, in 33 samples; PVM + PVS, in seven samples; PVM + PVS + PVY, in six samples; PLRV + PVM + PVY, in four samples; PVS + PVY, in two samples; PVM + PVP + PVY and PLRV + PVY, in one sample each. Therefore, PVM + PVY appeared to be the most frequent combination in mixed infections (approximately 59% of all the mixed infection cases). One in five samples contained up to three viruses, most often involving PVM and PVY. PVS was detected rarely and predominantly within triple combinations. The rates of mixed infections by year were as follows: 2021 – 44.2% (23 out of 52); 2022 – 50.0% (7 out of 14); 2023 – 78.6% (11 out of 14); and 2024 – 45.5% (15 out of 33). The highest rate was observed in 2023; however, the sample size for that year was limited (*n *= 14).

Statistical analysis using Pearson’s χ2 test showed that the differences in the rate of mixed infections across years (2021–2024) were statistically non-significant (χ2 = 6.94; *p *= 0.074), although there tended to be fluctuations (the highest values observed in 2023: 78.6%). For the PVM + PVY combination, significant differences between years were revealed (χ2 = 10.06;* p *= 0.018), whereas no statistical significance was observed for other combinations. Interregional comparison revealed significant differences (χ2 = 24.68;* p* < 0.001): the highest rate of mixed infections was observed in the Moscow (70%) and Sverdlovsk (76%) regions, while the lowest one was observed in the Astrakhan region (27%) and the “Other” category (24%). The “Other” category included regions with fewer than five samples, which were merged to meet the applicability requirements of the χ2 test. The rate of the PVM + PVY combination also differed significantly between regions (χ2 = 26.71; *p* < 0.001), indicative of prominent territorial heterogeneity in the distribution of this combination.

Mixed infections pose a serious threat, since the pathogenicity of individual viruses can increase substantially when a plant is simultaneously infected with two or more noncognate viruses [2]. Our findings underscore the high risk of coinfection in potato virus populations and the need to take that into account when performing epidemiological monitoring and designing effective plant protection strategies.

**The distribution and phylogenetic analysis of Potato virus Y (PVY)**

**Fig. 2:**
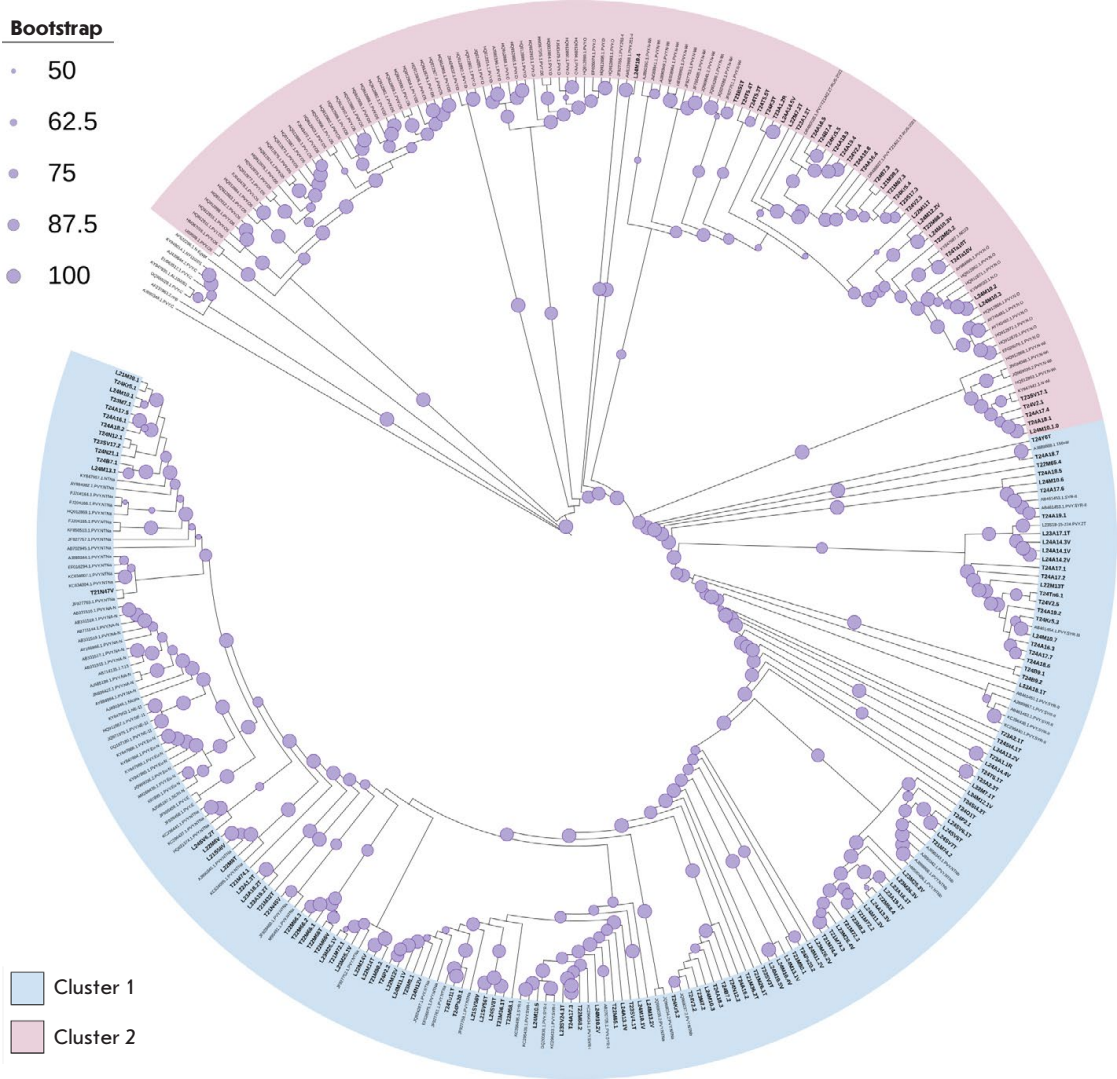
Phylogenetic relationship between *de novo *and reference-guided assembly PVY contigs identified in the Russian Federation and previously characterized PVY isolates. The maximum-likelihood phylogenetic tree was generated for complete nucleotide sequences. Bootstrap values for 1,000 replicates are indicated by the size of circles on branches (the smallest, 50–60%; the largest, 90–100%). Tip labels for the previously characterized PVY isolates show the GenBank accession number. Clusters with PVY variants belonging to the N-type are highlighted in blue (cluster 1), and variants belonging to the O-type are highlighted in pink (cluster 2). *De novo *contigs identified in this work are marked in bold

The phylogenetic analysis conducted using 158 assembled full-length PVY contigs, which included the complete PVY polyprotein sequence, together with 165 representative PVY isolates from GenBank, was characterized by significant diversity of PVY (Potato virus Y, *Potyvirus *genus, family *Potyviridae*) variants across the studied Russian regions (*Fig. 2*).

Previously, we [25] analyzed the RNA-seq data for the potato samples collected in 2021–2022 from only two regions: the Astrakhan region (Southern Federal District) and the Moscow region (Central Federal District), which showed that the NTNa and NTNb PVY recombinants were dominant. A significantly higher diversity in the PVY population was observed in the Astrakhan region, involving the additional recombinants N:O, N-Wi, SYR-I, SYR-II, SYR-III, and 261-4, whereas there were only five virus variants in the Moscow region: NTNa, NTNb, N:O, N-Wi, and SYR-I. These results allowed us to characterize the differences in the PVY population between the Southern and Central Federal Districts of the Russian Federation.

In this study, the geographic scope of sample collection was substantially expanded so that we could supplement our earlier findings with an analysis of new samples from various regions of the Russian Federation. The results for the two regions (Astrakhan and Moscow) obtained previously were integrated into a broader picture of PVY variant distribution across the Russian Federation.

**Fig. 3:**
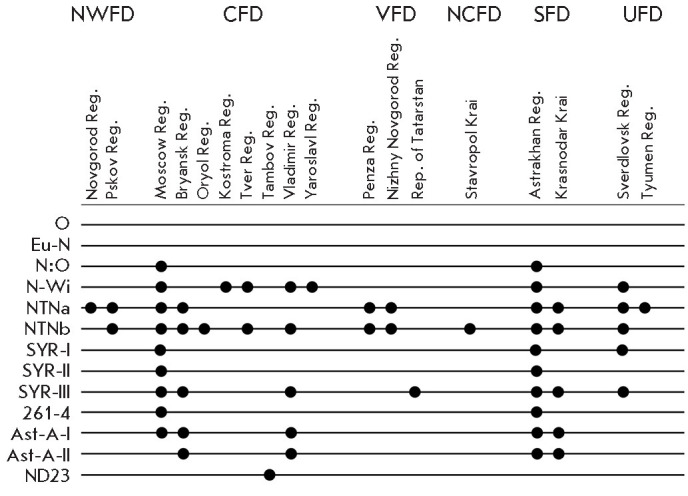
The geographic distribution of PVY recombinant variants identified in Russian regions. The regions are grouped by Federal Districts: NWFD – Northwestern, CFD – Central, VFD – Volga, NCFD – North Caucasian, SFD – Southern, and UFD – Ural Federal Districts. The names of the identified PVY recombinants are shown on the left. Dots indicate the presence of the corresponding PVY variant in the Russian region

The NTNa and NTNb recombinants were also shown to be dominant in almost all the studied regions. Moreover, only NTNa/NTNb recombinants were identified in the Pskov and Novgorod regions (Northwestern Federal District), Oryol and Penza regions (Central Federal District), Nizhny Novgorod region (Volga Federal District), Stavropol region (North Caucasus Federal District), and Tyumen region (Ural Federal District). In a 2021 study [11], an analysis of potato samples collected in different Russian regions revealed that NTNa was the main variant encountered. It is evident that over the past years, the NTNb variant has become as widespread across the Russian regions as the NTNa variant. Meanwhile, we identified only the N-Wi, N-Wi, and SYR-III variants, respectively (all belonging to the parental N type) in tuber samples collected from the Kostroma and Yaroslavl regions (Central Federal District) and the Republic of Tatarstan (Volga Federal District). The distribution of the N-Wi recombinant has also expanded recently (*Fig. 3*): we have detected it in samples collected in seven Russian regions.

A sequence related to the so-called rare recombinant PVY-ND23 was detected among the variants identified in our study [26]. Nucleotide sequence analysis of the T24Ta10 PVY variant from the Tambov region (Central Federal District) revealed a high degree of similarity to the GenBank isolate KY847997.1, which was identified in the United States in 2017. The identity percentage was as high as 99.36% with 99% coverage, which is indicative of a close phylogenetic relationship. The reference isolate has a N:O genotype, albeit with a shifted recombination breakpoint at the 5’ end of viral RNA. The constructed phylogenetic tree confirms clustering of the T24Ta10 variant having this genotype, suggesting a close evolutionary relationship with the aforementioned reference strain [27].

The highest diversity of recombinant PVY variants was revealed for the samples collected in the Astrakhan (Southern Federal District) and Moscow (Central Federal District) regions. Nearly all major clades were identified in these regions, including both the dominant recombinants NTNa, NTNb, N-Wi, and additional variants such as SYR-I, SYR-II, SYR-III, N:O, and 261-4 (according to our previous study [25] and the data obtained in 2023– 2024). However, the broad variability of the viral population observed in these regions may be partially attributed to the fact that the number of samples from these regions was the largest compared to other territories.

When analyzing the RNA-seq data for the samples collected in the Astrakhan region in 2021–2022, we first described two novel PVY recombinants: Ast-A-I and Ast-A-II [16]. Contigs corresponding to these recombinant PVY variants were also obtained by analyzing tuber samples collected in the Astrakhan region in 2023–2024. Interestingly, these variants were also detected in tuber samples from the Bryansk and Vladimir regions (Central Federal District) and the Krasnodar Krai (Southern Federal District) in 2024, attesting to the fact that they have become established in the PVY population.

Hence, the phylogenetic analysis revealed that two stable phylogenetic PVY lineages circulate in the studied Russian regions. The first lineage is represented by dominant recombinant variants belonging to the parental type N, while the second, more diverse group includes recombinant variants belonging to the parental type O. No variants belonging to the types PVY-C, PVY-O5, and PVY-NA-N were detected in our study. The presence of rare variants (Ast-A-I, Ast-A-II, 261-4, and ND23) may be indicative of both a high level of evolutionary dynamics within the viral population (*Fig. 3*) and high likelihood that these PVY variants have been introduced via seed material.

Earlier studies focusing on PVY variants in different Russian regions have also suggested the widespread distribution of this virus [8, 9, 10, 11, 12]. While the samples collected from commercial fields in 2015– 2018 contained the parental non-recombinant strain PVYO along with NTN [9], in subsequent years, only PVY recombinant variants (NTNa, N:O, and N-Wi) were identified in the Northwestern, Volga, and Far Eastern Federal Districts; SYR-I, SYR-II, and 261-4 recombinant variants, in the Volga Federal District [11]; and NTNa, SYR-III, and 261-4 recombinant variant, in the Novosibirsk region [12].

Overall, the diversity of PVY recombinant variants has noticeably broadened in recent years. The recombinant variant 261-4 is a notable example. It was originally classified as rare [27] and was first detected in the Russian Federation in the Far Eastern Federal District [11]. More recently, this recombinant variant has been encountered in the Novosibirsk region [12], as well as in the Moscow and Astrakhan regions (by our research team).

**Phylogenetic analysis and geographic distribution of Potato viruses M, S, and P**

**Fig. 4:**
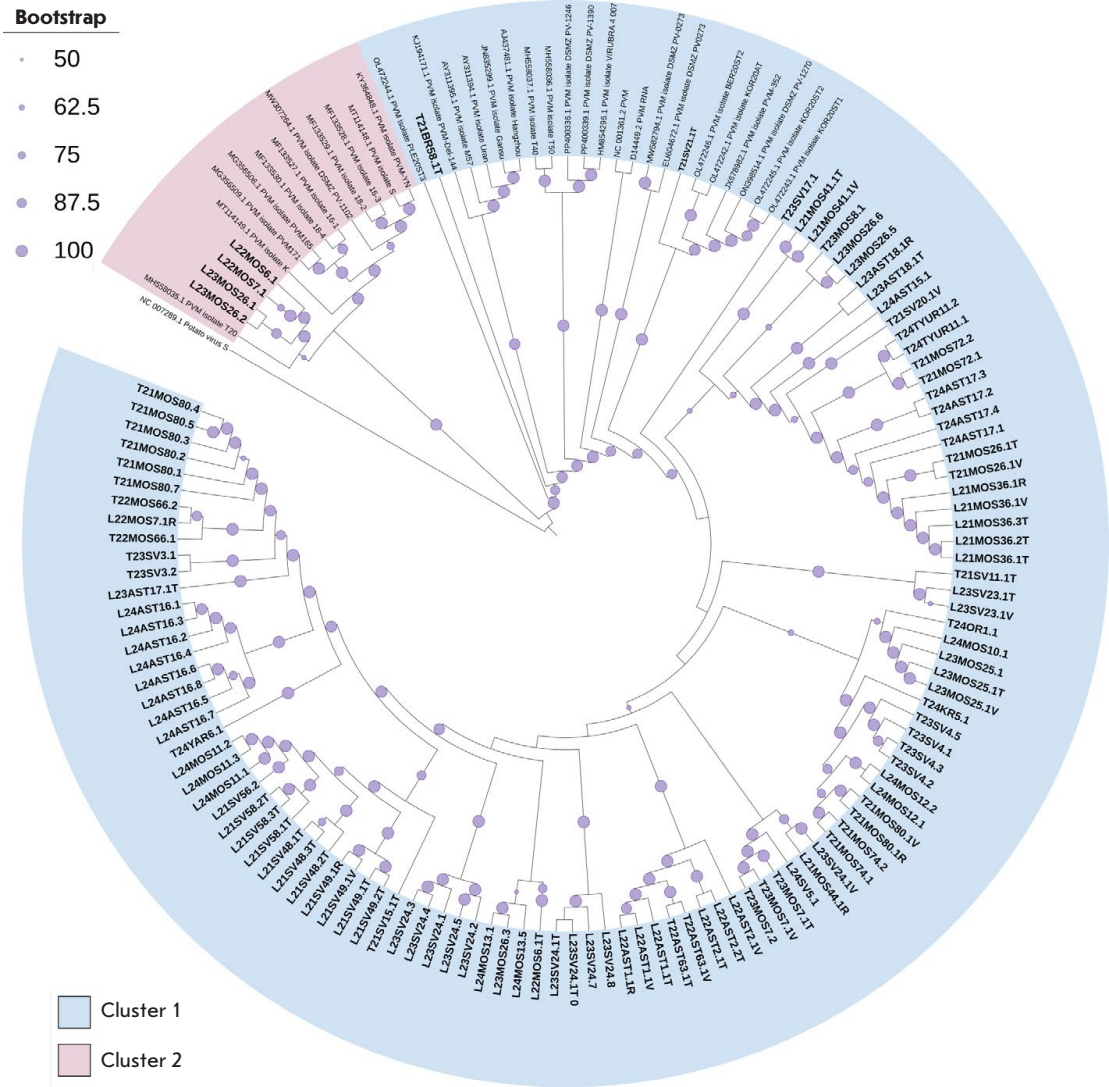
Phylogenetic relationship between *de novo *PVM contigs identified in the Russian Federation and previously characterized PVM isolates. The maximum-likelihood phylogenetic tree was generated for complete nucleotide sequences. Bootstrap values for 1,000 replicates are indicated by the size of circles on branches (the smallest, 50–60%; the largest, 90–100%). Tip labels for the previously characterized PVM isolates show the GenBank accession number.* De novo *contigs identified in this work are marked in bold

**Fig. 5:**
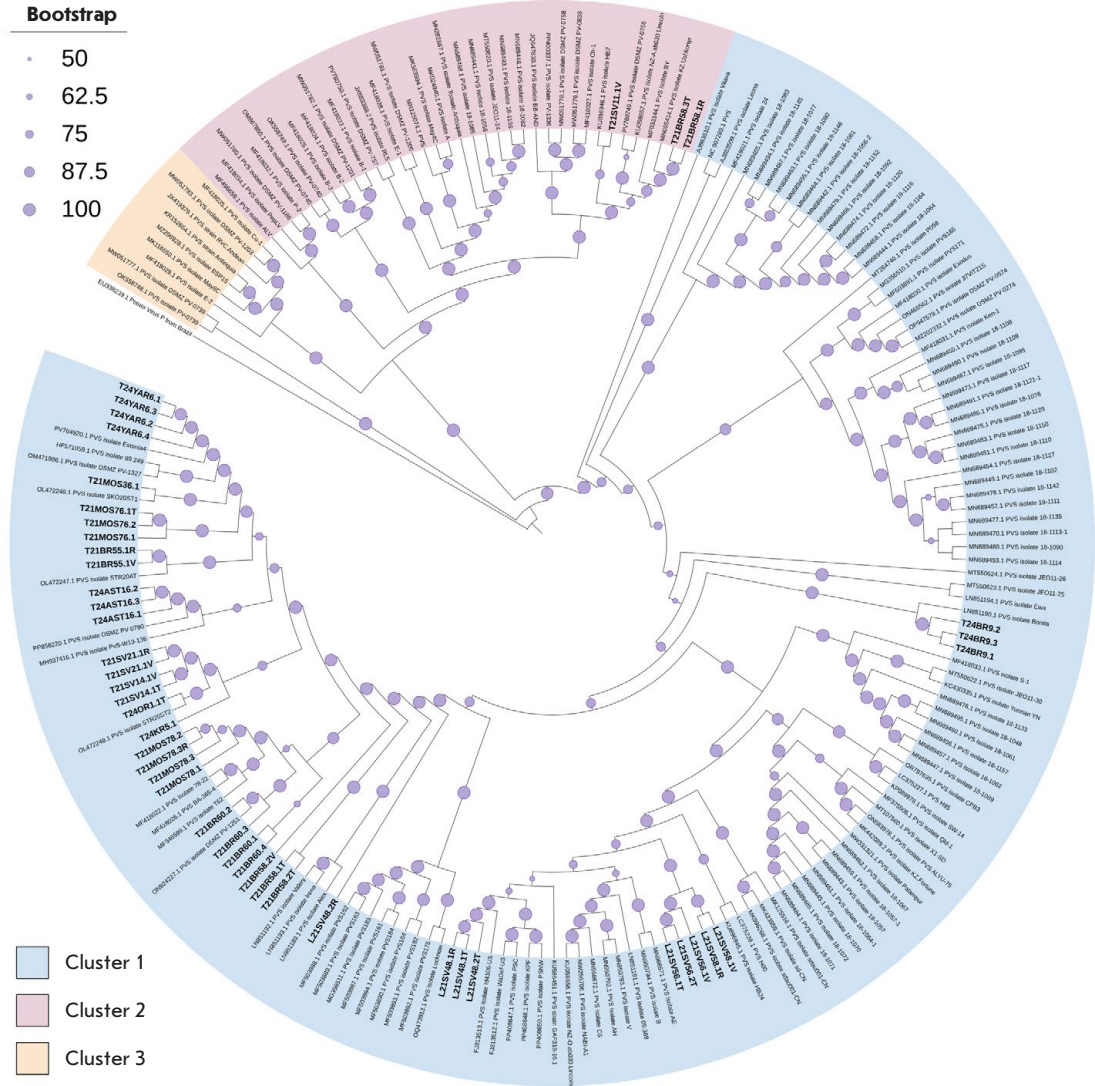
Phylogenetic relationship between *de novo *PVS contigs identified in the Russian Federation and previously characterized PVS isolates. The maximum-likelihood phylogenetic tree was generated for complete nucleotide sequences. Bootstrap values for 1,000 replicates are indicated by the size of circles on branches (the smallest, 50–60%; the largest, 90–100%). Tip labels for the previously characterized PVS isolates show the GenBank accession number. *De novo *contigs identified in this work are marked in bold

We conducted a phylogenetic analysis of the identified full-genome contigs of the potato viruses M and S by comparing them with the full-genome sequences of cognate viruses available in the NCBI GenBank database (*Fig. 4*, *Fig. 5*).

The phylogenetic tree constructed using the analyzed sequences of strains/isolates of Potato virus M (PVM, *Carlavirus *genus, family *Betaflexiviridae*) contains two clusters: Cluster 1 (highlighted in blue) and Cluster 2 (pink), indicating that there are two major phylogenetic lineages (*Fig. 4*). Cluster 1 comprises most of the sequences and is characterized by high intragroup variability, which may be indicative of either a wide geographic distribution or long-term viral evolution within the population. This cluster includes our sequences detected in the samples from different Russian regions, including the Moscow, Yaroslavl, and Oryol regions (Central Federal District), the Astrakhan region and Krasnodar Krai (Southern Federal District), as well as the Sverdlovsk and Tyumen regions (Ural Federal District). Cluster 2 includes only contigs from samples collected in the Moscow region (Central Federal District) and nucleotide sequences of the genomes of PVM from Slovakia, Canada, and Germany. Within this cluster, the PVM sequences identified by us form a branch most closely related to sequences from Slovakia, which is indicative of a common source or recent genetic exchange between populations. Of particular interest is the PVM contig sequence identified in a sample from the Bryansk region (Central Federal District), which occupies an intermediate position between the two clusters. A similar position is occupied by the GenBank sequence OL472244.1 from Slovenia, potentially indicative of analogous recombination events or genetic exchange between populations that led to the formation of these intermediate genotypes.

The nomenclature of Potato virus S (PVS,* Carlavirus *genus, family *Betaflexiviridae*) involves three main phylogenetic groups: PVSI, PVSII, and PVSIII [28, 29]. Isolates of the phylogroup PVSI (formerly known as PVSO) are widely distributed worldwide, whereas PVSII isolates (formerly known as PVSA) are more commonly found in Chile, Colombia, and Brazil [30, 31]. Furthermore, a third phylogroup, PVSIII, confined to the Andean region of South America (primarily Colombia), has been identified [28]. In our study, most of the PVS contigs identified in the Moscow, Bryansk, and Oryol regions (Central Federal District), the Astrakhan region and Krasnodar Krai (Southern Federal District), and the Sverdlovsk region (Ural Federal District) of the Russian Federation were assigned to the phylogenetic branch of the PVSI strain (*Fig. 5*, Cluster 1). Meanwhile, the phylogenetic analysis showed that two contigs from tubers collected in the Sverdlovsk (Ural Federal District) and Bryansk (Central Federal District) regions cluster together with isolates of the PVSII strain: that is the first confirmed detection of this strain in the Russian Federation (*Fig. 5*, Cluster 2). PVSO (PVSI) isolates generally do not induce any prominent symptoms in most commercial potato cultivars, and this fact facilitates their cryptic transmission via seed material. In contrast, PVSA (PVSII) isolates are more pathogenic and can cause substantial crop yield loss, which increases their phytosanitary significance for potato production in the Russian Federation. Our findings indicate that there are two PVS variants belonging to two main phylogenetic lineages (PVSI and PVSII) in the samples. The PVSIII variant was not detected in Russian samples (*Fig. 5*, Cluster 3).

We detected Potato virus P (PVP, *Carlavirus *genus, family *Betaflexiviridae*) in a potato tuber sample from the Sverdlovsk region (Ural Federal District). This virus is rarely found in Russia and is not considered economically significant compared to PVY, PVS, and PVM. Nevertheless, its detection is of interest for monitoring viral infections of potato and assessing the phytosanitary status of seed material. PVP was first detected in Brazil and Argentina [32]; for a long time, it was considered to be confined to the South American region. The symptoms of potato plants affected by PVP are mild and remain insufficiently characterized [32]. In the Russian Federation, PVP was first identified in 2018 [10]. Later, in 2021, cases of PVP infection in potato were primarily reported in the Far Eastern Federal District and, to a lesser extent, in the Northwestern Federal District [11]. However, the impact of this virus on potato crop yield has not been assessed yet, since the symptoms induced by the Russian PVP isolate still remain to be characterized. The full-length genome of PVP assembled by us is 8,394 nucleotides long. A comparative analysis revealed a high degree of similarity with the Russian isolate published earlier: 97.89% nucleotide sequence identity at 100% coverage (GenBank: LC480818.1) [10]. In contrast, the level of identity with a Brazilian isolate was 77.85% at 85% coverage. These data confirm that the sample belongs to the Russian population of PVP, allowing one to classify it as a regional isolate.

**The distribution and phylogenetic analysis of the Potato leafroll virus**

The complete nucleotide sequence of the coding sequence (CDS) of Potato leafroll virus (PLRV,* Polerovirus *genus, family *Solemoviridae*) in potato plant samples was for the first time identified and characterized by high-throughput sequencing in the Russian Federation. The virus was detected in potato plant samples from the Moscow (Central Federal District) and Novgorod regions (Northwestern Federal District). In the Moscow region, PLRV was detected in different categories of potato seed material. Specifically, PLRV was detected in the leaves and tubers of first-generation plants, as well as in the leaves of plants classified as super-super-elite, indicating the potential presence of the virus even in high-reproductive categories of seed potatoes. Overall, five complete PLRV CDS sequences were obtained from the samples collected in the Moscow region. Additionally, one complete CDS sequence was obtained from a tuber sample from the Novgorod region.

**Fig. 6:**
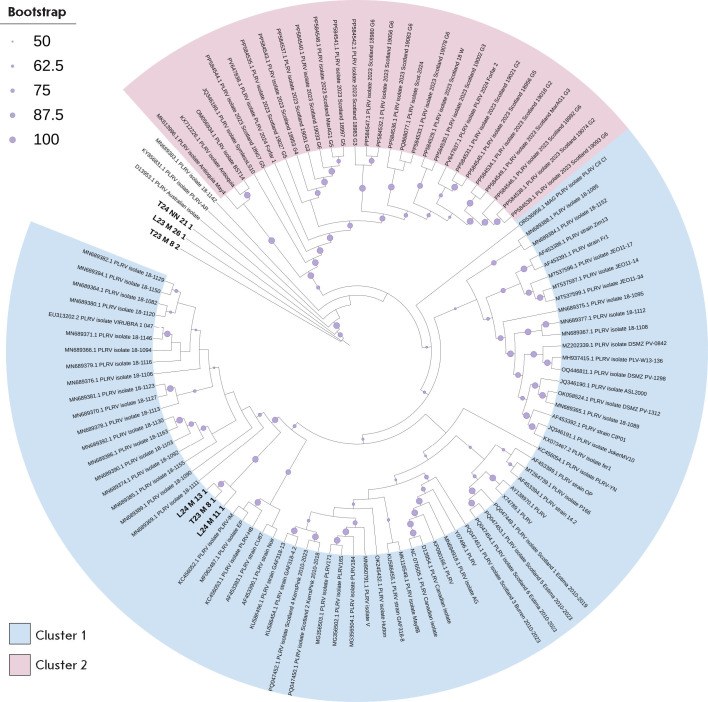
Phylogenetic relationship between de novo PLRV contigs identified in the Russian Federation and previously characterized PLRV isolates. The maximum-likelihood phylogenetic tree was generated for complete nucleotide sequences. Bootstrap values for 1,000 replicates are indicated by the size of circles on branches (the smallest, 50–60%; the largest, 90–100%). Tip labels for the previously characterized PLRV isolates show the GenBank accession number.* De novo *contigs identified in this work are marked in bold

Phylogenetic analysis and pairwise genetic distance computation revealed that the identified PLRV variants form two distinct phylogroups (*Fig. 6*). Cluster 1 involves variants T23_M_8_2, L23_M_26_1, and T24_NN_21_1, collected from the Moscow and Novgorod regions. These variants exhibit a high degree of similarity to each other (0.0009–0.0239), form an independent minicluster, and may represent a local sublineage. Comparison of our contigs to the sequences from other databases showed that these variants are most closely related to Argentinian isolates (e.g., GenBank: KY856831) and Australian PLRV genomes (e.g., isolate D13953.1, GenBank). Cluster 2 involves all the variants from the Moscow region: T23_M_8_1, L24_M_11_1, and L24_M_13_1, which are completely identical to each other but differ significantly from the isolates in Cluster 1 (~ 0.0594). The genomes most closely related to our samples were those from the

MN68937x–MN68939x series (GenBank), deposited as isolates from Kenya. The second major branch of Cluster 2 includes individual genomes from Asia and Europe (e.g., the EF and HQ series from China, India, the Netherlands, etc., GenBank). This group is phylogenetically distant from our variants and forms an independent evolutionary lineage that is not directly related to the African branch. This distribution suggests that some of our isolates belong to a phylogenetic lineage represented by isolates circulating in East Africa, while others are distinct and reside near the branches corresponding to PLRV isolates from Argentina and Australia, which, potentially, can be indication that there exist multiple independent pathways for virus introduction.

## CONCLUSIONS

We have conducted a large-scale study focusing on the distribution and genetic composition of populations of major economically important potato viruses across 18 regions in six federal districts of the Russian Federation. The research was carried out using potato samples of high-seed generations, originally derived from sanitized (virus-free) seed potatoes grown on commercial fields under conditions of natural infection by aphid vectors transmitting the main potato viruses. We have demonstrated widespread distribution only of the recombinant PVY variants, including NTNa, NTNb, N-Wi, N:O, SYR-I, SYR-II, SYR-III, and 261-4, most of which cause the potato tuber necrotic ringspot disease. Sporadic cases of another dangerous virus, PLRV, were also detected, along with PVY. PVM and PVS were identified in many regions; PVS was represented not only by weakly pathogenic variants belonging to the PVSI phylogroup, but also by PVSII variants exhibiting significant pathogenicity. Importantly, many viruses were detected in both potato leaf samples and tubers, suggesting that they had been transmitted to the subsequent plant generations through seed material. The observed geographic variations can be attributed to the seasonal and climatic characteristics of the regions, as well as to the varietal composition of plantings, which needs a separate analysis. Of particular note, the molecular diversity of potato viruses and their distribution across the Russian Federation increase annually; variants exhibiting higher pathogenicity emerge. Our findings underscore the importance of regular monitoring of the viral population and rigorous adherence to phytosanitary regulations, as well as the importance of developing new strategies for managing viral infections in potato.

## Abbreviations

PVY: Potato virus Y
PVS: Potato virus S
PVM: Potato virus M
PVP: Potato virus P
PLRV: Potato leafroll virus
dsRNA: double-stranded RNA
RF: Russian Federation
CFD: Central Federal District
NWFD: Northwestern Federal District
VFD: Volga Federal District
NCFD: North Caucasian Federal District
SFD: Southern Federal District
UFD: Ural Federal District
FEFD: Far Eastern Federal District
